# Applications of array technology: identification of molecular targets in bladder cancer

**DOI:** 10.1038/sj.bjc.6601406

**Published:** 2003-12-09

**Authors:** M Sánchez-Carbayo, C Cordon-Cardo

**Affiliations:** 1 Division of Molecular Pathology, Memorial Sloan-Kettering Cancer Center, 1275 York Avenue, New York, 10021 NY, USA

**Keywords:** bladder cancer, molecular target, DNA microarrays, tissue arrays

## Abstract

High-throughput microarrays are being used in expression profiling analyses with the objecties of gene and pathway discovery, functional characterization of genes, and tumor subclassification. This review summarizes bladder cancer studies dealing with both *in vitro* models and clinical specimens, using distinct microarray platforms for target discovery.

AQMicroarrays constitute a group of technologies characterised by the common availability of measuring hundreds or thousands of items, including DNA sequences, RNA transcripts or proteins, within a single experiment using miniaturised devices. The appropriate experimental design and the use of well-characterised *in vitro*, *in vivo* systems, as well as well-annotated clinical specimens allow detailed analysis of biological and clinical phenotypes. Thus, array technologies represent high-throughput means to identify molecular targets associated with these biological and clinical phenotypes by comparing samples representative of distinct disease states.

Hybridisation-based methods and the microarray format constitute together an extremely versatile platform provide for both static and dynamic views of DNA structure, as well as RNA and protein expression patterns in cultured cancer cells and tumour tissues. The most widespread use of this technology to date has been the analysis of gene expression (Duggan *et al*, 1999; Lipshutz *et al*, 1999). There is an increasingly broad range of additional applications for microarrays, including genotyping polymorphisms and mutations (Hacia and Collins, 1999; Fan *et al*, 2000), determining the sites of DNA-binding proteins (Iyer *et al*, 2001), and identifying structural alterations using arrayed comparative genome hybridisation (CGH) (Pinkel *et al*, 1998). Similar to other tumours (De Risi *et al*, 1996; Golub *et al*, 1999; Sorlie *et al*, 2001; van 't Veer *et al*, 2002), it is expected that the histopathological diagnosis of bladder cancer will be complemented by these comprehensive procedures. It is also expected that such studies will generate further insights regarding optimal treatments, based on the individual molecular signatures of patients affected by bladder cancer.

The development and implementation of high-throughput array technologies to primary human tumours is changing the scientific and clinical paradigm, providing novel predictive and therapeutic targets for the cancer patient. The challenge now resides in integrating the anatomic, mostly descriptive knowledge of neoplastic disease processes, with cellular and molecular biology, as well as genetics. To the simplicity of the study of a single molecular marker, we may be able to integrate the complexity of multiple biological determinants, participating in signalling pathways and biological networks. Observational measurements will become quantified, and these values may guide predictive nomograms for appropriate intervention. Comprehensive information may, in turn, modify empirical clinical treatments for mechanism-based therapies, aimed at individualised approaches for the cancer patient.

These technologies represent optimal tools for target identification at initiation and critical steps along bladder cancer progression. Focus should be directed towards the elucidation of the molecular pathways involved in bladder tumorigenesis, and progression into invasive and metastatic disease. Further research is also warranted to characterise the phenomena of squamous metaplasia and squamous carcinoma of the bladder, as well as squamous differentiation in the background of transitional cell carcinoma. The analysis of clinical specimens is expected to provide expression patterns that may allow to identify which patients would respond to intravesical or systemic treatments, or present a higher progression risk to develop invasive or metastatic disease. These studies will result in individualised bladder cancer targeted therapies and development of biomarkers of diagnostic and predictive value for patients with bladder cancer.

## MOLECULAR TARGET IDENTIFICATION IN BLADDER CANCER USING EXPRESSION PROFILING WITH DNA MICROARRAYS

**Figure 1:**
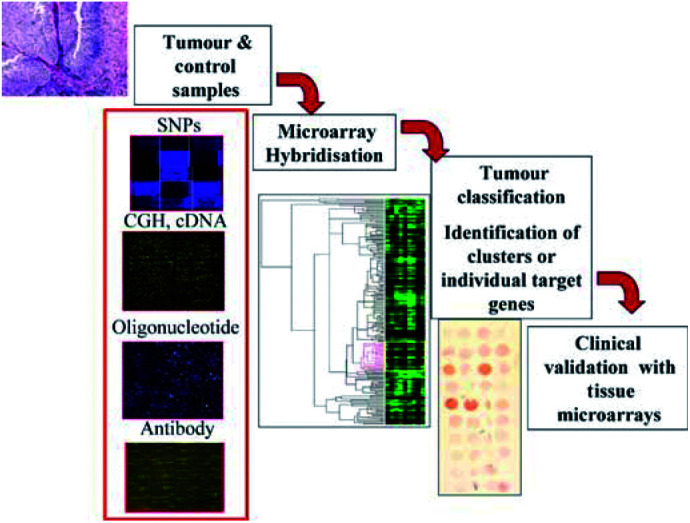
General scheme of the procedure used in tumour expression profiling for target identification and validation. RNA isolation from cell lines, tumour biopsy, and control samples is followed by labeling of the probe, hybridisation with the DNA microarray, data acquisition, and analysis. Verification of the results can be performed using different approaches, such as tissue microarray analysis.

The main challenge in studies using high-throughput technologies resides in making efficient and maximal use of the generated information (Figure 1

). The amount and complexity of data require multiple computational genomic approaches, including bioinformatic and biostatistical analyses. Molecular relationships need to be established with normal and distinct diseased states. In order to bring this knowledge into clinical settings, it will be critical to validate the relevant molecular determinants, and to include them as part of the ongoing clinical trials. Microarray-based gene expression profiling to the study of bladder cancer can be divided into those analyzing *in vitro* systems, and those centering on clinical material, with the objectives of achieving gene and pathway discovery, functional classification of genes, and a new classification based on tumour subtypes.

## MOLECULAR STUDIES AIMED AT GENE AND PATHWAY DISCOVERY

These analyses are mainly based on functional association of changes in gene expression between different cell states or phenotypes. This use of associating a change in the expression of a gene with a change in physiological state is one the simplest ways in which gene expression profiling can be used to suggest or predict gene function. Alternatively, expression profiling can be used for the functional classification of genes, as it is often referred to as ‘guilt by association’. This method is based on the observation that genes with related expression patterns, genes that presumably are coregulated, are likely to be functionally related and involved in the same biological processes or physiological pathways. When genes with similar expression profiles are grouped, a process referred to as clustering, novel genes (usually ESTs) are often found mixed with genes of known function.

## MOLECULAR STUDIES AIMED AT FUNCTIONAL CLASSIFICATION OF GENES

This represents the traditional approach of assigning a functional role to a gene when overexpressed, and observing the effect(s) of its expression on known pathways or processes. Such an approach has been especially useful in identifying the downstream targets of transcription factors. The genes identified as either up- or downregulated in these experiments are likely to play important roles in the signalling network in which the gene under investigation participates.

## MOLECULAR STUDIES AIMED AT TUMOUR SUBCLASSIFICATION

This is one of the most promising and powerful applications of expression profiling with expression microarrays. The integration of gene expression patterns is providing complementary tools to histopathological criteria for classifying tumours into biologically meaningful and clinically useful categories. In addition, expression profiling of well-annotated tumour specimens has the potential of identifying target genes for novel diagnostic, prognostic or therapeutic approaches. High-throughput transcriptome analysis will become a means at improving cancer treatment by an early and accurate diagnosis of tumour subtype and determining the most effective therapeutic intervention.

## BLADDER CANCER STUDIES USING *IN VITRO* MODELS

Expression profiling using bladder cancer cell lines has been used to gain insight into the molecular events associated with clinical disease states, assigning potential functional roles to novel genes in both tumorigenic and tumour progression processes. The following study represents an example of how the technology can be applied to *gene and pathway discovery* in bladder cancer. Tumour cell growth inhibition mediated by genistein was produced to the susceptible bladder tumour line TCCSUP. Expression profiling was then analysed at various time points, using cDNA chips. Induction of genes involved in cell growth and cell cycle, such as EGR-1, was observed, and these events were related to the proliferation and differentiation effects of treatment (Chen *et al*, 2001). The gene induction in T24 invasive bladder cancer cells exposed to 5-aza-2′-deoxycytidine has also been monitored by expression profiling studies using oligonucleotide microarrays (Liang *et al*, 2002). This analysis revealed the relevance of interferon gamma signaling in the effects of this methylation inhibitor treatment.

An example of the *functional classification of genes* applied to bladder cancer is the study comparing the expression patterns of p53-mediated apoptosis in resistant tumour cell lines *vs* sensitive tumour cell lines using cDNA arrays. The ECV-304 bladder carcinoma cell line was selected for resistance to p53 by repeated infections with a p53 recombinant adenovirus Ad5CMV-p53. A number of potential p53 transcription or related targets were identified, playing roles in cell cycle regulation, DNA repair, redox control, cell adhesion, apoptosis, and differentiation. Proline oxidase, a mitochondrial enzyme involved in the proline/pyrroline-5-carboxylate redox cycle, was identified upregulated in sensitive cells, but not in resistant ones. Further experiments showed the implication of proline oxidase and the proline/P5C pathway in p53-induced growth suppression and apoptosis (Maxwell and Davies, 2000). The expression patterns of a metastatic variant cell line, the so-called T24T, and the invasive bladder cancer cell line T24 have been studied using oligonucleotide microarrays. The functional significance of the genetic differences of these cell lines can be assessed by means of positional expression profiling methods that compare the expression data generated by oligonucleotide microarrays based upon chromosomal position (Harding *et al*, 2002). The combined use of spectral karyotyping (SKY) and CGH and expression profiles of these cells provided high correlation for certain chromosomes such as 8, 12 and X. This multimodal approach allowed the validation of candidate genes involved in bladder progression, such as RhoGDI2 (chromosome 12) at the genetic level (Harding *et al*, 2002).

*Tumour subtypes classification* of bladder cancer cell lines can also be achieved by means of gene expression analyses. The expression profiling of nine bladder cancer cell lines have been compared against a pool containing equal RNA quantities of each of them using cDNA arrays (Sanchez-Carbayo *et al*, 2002). Hierarchical clustering classified these tumour cells according to the histopathological characteristics of the tumours they were derived from. Caveolin-1 and keratin 10 were differentially expressed in a squamous carcinoma cell line and certain invasive tumour cell lines, when compared to cells derived from a papillary superficial bladder tumour. Interestingly, the expression of these genes in primary bladder tumours spotted on tissue microarrays was significantly associated with squamous differentiation, histopathological stage, and tumour grade. Additionally, when a bootstrapping resampling technique was applied on hierarchical clustering, the cells clustered based on their p53, RB, and INK4A status. E-cadherin, zyxin and moesin were identified as genes differentially expressed in these clusters. It is noteworthy that the expression of these genes was significantly associated with histopathological stage and tumour grade as well. These results revealed that molecular profiling clustered bladder cancer based on histopathogenesis and biological criteria. Moreover, gene profiling identified novel biomarkers of the disease that were proven to be associated with clinical and histopathological variables when validated on tumour specimens using tissue microarrays at the protein level, using well-characterised antibodies and immunohistochemistry (Sanchez-Carbayo *et al*, 2002).

## BLADDER CANCER STUDIES USING CLINICAL SPECIMENS

Microarray analyses have been used to correlate changes in the expression of specific genes and groups of genes within distinct bladder tumours. Following the biological validation of these expression–phenotype correlations, the result will be a more complete list of the genes controlling cancer development and progression. Gene expression profiling of bladder cancer tissues have identified signature genes that robustly distinguish bladder cancer subclasses. Such signature genes would ideally provide a molecular basis for classification, yielding insight into the molecular events underlying different clinical bladder cancer phenotypes.

There have been few studies dealing with molecular classification of bladder cancer expression profiling using DNA microarrays. The first report monitored the expression patterns of superficial and invasive tumour cell suspensions prepared from 36 normal and 29 bladder tumour biopsies using oligonucleotide microarrays. This study also analysed pools of cells made from normal urothelium, as well as pools of tumours of different stages, such as from pTA grade I and II and pT2 grade III and IV bladder cancer specimens (Thykjaer *et al*, 2001). Hierarchical clustering of gene expression levels grouped bladder cancer specimens based on tumour stage and grade. By organising genes with similar expression patterns into clusters, several functionally related genes were identified. By examining log-fold changes of expression, the most significant genes included those involved in cell cycle, cell growth, immunology, cell adhesion, transcription, and protein metabolism. Superficial papillary tumours showed increased transcription factor and ribosomal levels, as well as proteinase encoding genes upregulation. In the invasive tumours, increased levels of cell cycle, growth factor networks related and oncogene transcripts were observed. A loss of cellular adhesion genes was found in invasive tumours, which may in turn be related to invasion and metastasis. The invading tumour cells seem to challenge the immune system, as reflected by an increase in immunology-related proteins (Thykjaer *et al*, 2001).

The combination of separate expression profiling studies of bladder tumours and bladder cancer cell lines has allowed the identification of the tumour-suppressor role of KiSS-1 in bladder cancer progression (Sanchez-Carbayo *et al*, 2003a,2003b). Lower transcript levels of KiSS-1 were observed in bladder carcinomas, as compared to superficial tumours, and these ratios provided prognostic information. Lower expression of this gene was also observed in cells derived from advanced bladder tumours (Sanchez-Carbayo *et al*, 2002). The analysis of the expression patterns of KiSS-1 by *in situ* hybridisation on tissue microarrays confirmed the loss of KiSS-1 in the progression of the disease, and was associated with tumour stage, grade, and overall survival. In this example, gene expression profiling identified a novel target involved in bladder cancer progression with clinical relevance (Sanchez-Carbayo *et al*, 2003a,2003b).

The most extensive expression profiling study of bladder tumours reported to date has dealt with the development of a predictive classifier of Ta, T1, and T2+ bladder carcinoma subclasses. The use of a support vector machine algorithm allowed prediction of these tumour subclasses with 75% accuracy, in an independent set of patients. This report revealed the diagnostic and prognostic potential of bladder tumour profiling using cross-validation strategies to evaluate the clinical impact of the classifier defined using independent series of tumours. Smad6 and cyclin G2 were also identified as Ta/T1 classifier genes, and their immunostaining patterns were validated on tissue microarrays by immunohistochemistry (Dyrskjot *et al*, 2003). This report classified bladder tumours based on current histopathological schemes, representing the first attempt to predict recurrence within 2 years for patients with bladder cancer.

A recent study has compared the expression profiles of early-stage and advanced bladder tumours using cDNA microarrays. Gene profiling successfully classified bladder tumours based on their progression and clinical outcome. The application of bootstrapping techniques to hierarchical clustering and multidimensional analyses segregated early-stage and invasive transitional carcinomas, and identified early-stage tumours showing gene profiles similar to invasive disease. More importantly, it separated carcinoma *in situ* from papillary superficial lesions and subgroups within early-stage and invasive tumours displaying different overall survival. Molecular biomarkers of potential clinical significance and critical molecular targets associated with bladder cancer progression were identified using different techniques, including standard *t*-test, single-gene logistic regression, and support vector machine algorithms. For example, p33ING1 was found to be significantly associated with pathological stage, tumour grade, and overall survival, when validated by immunohistochemistry using tissue microarrays. Analysis of the annotation of the most significant genes revealed the relevance of critical genes and pathways during bladder cancer progression (Sanchez-Carbayo *et al*, 2003b).

The application of tissue microarrays represents a high-throughput approach for validation of potential novel markers for bladder cancer by immunohistochemistry or *in situ* hybridisation in paraffin blocks (Kononen *et al*, 1998; Schraml *et al*, 1999; Richter *et al*, 2000; Nocito *et al*, 2001). Several reports describe the rapid evaluation of targets of interest, such as cytoskeletal actin-associated gelsolin or E-cadherin (Rao *et al*, 2002), Na,K-ATPase or cell-cycle-related markers (Espineda *et al*, 2003). The most extensive tissue microarray in bladder cancer, analysing over 2000 bladder carcinomas, revealed the prognostic utility of cyclin E (Richter *et al*, 2000). Focus is intensified within this field to automate the construction of tissue microarrays. This high-throughput approach allows further characterisation of novel genes by *in situ* hybridisation of ESTs and known genes, when specific antibodies are not available to study their potential clinical relevance.

## MOLECULAR STUDIES USING MICROARRAY PLATFORMS FOR TARGET DISCOVERY

In addition to transcriptome expression microarrays, specific oligonucleotide microarrays have been applied to the study of DNA variation in clinical material. Multiple probes of short length that differ in sequence at a single base have been designed to identify simple polymorphisms and allelic variations in DNA. The primary applications of these types of microarrays have dealt with automated high-throughput identification of mutations in critical genes such as TP53, a valuable predictor for bladder cancer outcome (Lu *et al*, 2002), and in the genotyping of single-nucleotide polymorphisms (SNPs) (Primdahl *et al*, 2002). Not only SNP arrays confirm known areas of chromosomal losses, they may also have identified areas with common allelic imbalances that could harbour potential tumour suppressors involved in bladder cancer progression (Primdahl *et al*, 2002). Although initial chips were restricted to the polymorphic areas contained in the arrays, in the future, it should be possible to fabricate high-density SNP microarrays for other predefined chromosomal locations or larger regions, which could make unknown areas informative.

The application of high-throughput CGH arrays will confirm the alterations found at the genomic level in a comprehensive detailed manner (Pinkel *et al*, 1998). Microarrays can be used to define gene copy number changes based on cohybridisation of labelled experimental and normal DNA to an array of genomic DNA. This technique is well suited to high-throughput whole genome detection of chromosomal gains and losses at high resolution, enabling rapid detection of homozygous loss, which allow molecular phenotyping of tumours based on the underlying abnormalities and the detection of amplicons, which may be associated with overexpression of oncogenes. This provides a significant advantage over laborious pregenome mapping and transcript identification strategies. An additional advantage of this approach is that probes may be generated using paraffin-embedded material, greatly expanding the available specimens for analysis. This feature can allow study of genetic changes in tumour progression (Veltman *et al*, 2003).

It should be noted that microarrays technology is a convenient platform for assays involving biomolecules other than nucleic acids. Arrays of tissues, peptides, antibodies, proteins, and even cells have been developed (MacBeath and Schreiber, 2000; Haab *et al*, 2001; Reineke *et al*, 2001; Ziauddin and Sabatini, 2001). This is further evidence of the strength and versatility for high throughput screening. Protein microarrays provide means of rapidly validating the genes identified by expression profiling using DNA microarrays, at the protein level.

## DISCUSSION

The majority of the studies reported to date have utilised the DNA microarray platform using both oligonucleotide and cDNA formats. On one side, the commercial oligonucleotide arrays, mainly those manufactured by Affymetrix, have been used in most of the studies using clinical material. The Rosetta oligonucleotide arrays, well known through the predictive studies performed in breast tumours (van 't Veer *et al*, 2002), have not been utilised in bladder cancer yet. On the other side, the homemade cDNA microarrays have been utilised in most of the *in vitro* studies reported in bladder cancer. Further studies are required using different formats and platforms to consolidate the clinical relevance of the findings recently reported. The majority of the studies using clinical material have utilised standard commercial hybridisation protocols (Thykjaer *et al*, 2001; Dyrskjot *et al*, 2003), although the potential of linear amplification hybridisation protocol has also been described (Sanchez-Carbayo *et al*, 2003a,2003b). Many data analysis tools have been described for target identification. They vary from *t*-test algorithms or standard hierarchical clustering (Thykjaer *et al*, 2001) to single logistic regression analysis, to take into account the dispersion of the data when comparing also two categories, or the application of bootstrapping techniques to evaluate the robustness of hierarchical clustering (Sanchez-Carbayo *et al*, 2003b). Bipartition of the data into training and validation sets and the use of supervised vector machine algorithms with or without factor analysis have been utilised for subtype classification (Sanchez-Carbayo *et al*, 2003b) and predictive purposes (Dyrskjot *et al*, 2003).

As compared to other solid tumours, there are few reports using DNA microarrays. The use of the technology using *in vitro* models is limited and warrants further studies in each of the objectives presented here as gene and pathway discovery, functional classification of genes, and tumour subclassification. The first two objectives represent an open field depending on the research area of laboratories focusing on bladder cancer. Many targets have been identified to be involved in bladder cancer progression, and comprehensive study of the mechanisms by which these molecules are involved in bladder tumorigenesis or progression might contribute to novel therapies or diagnostic tools for bladder cancer. Interestingly, no *in vivo* study focused on bladder cancer using DNA microarrays has been reported to date. Most of the bladder cancer cell lines commercially available have been studied with the aim of identification of genes related to histopathological subtypes (Sanchez-Carbayo *et al*, 2002). However, further studies are warranted, with new cell lines and other DNA platforms.

Bladder cancer studies using clinical material are also relatively limited by two studies using the oligonucleotide format (Thykjaer *et al*, 2001; Dyrskjot *et al*, 2003), and another study using cDNA microarrays (Sanchez-Carbayo *et al*, 2003b). These reports have focused mainly on tumour classification, finding satisfactory results to segregate superficial and invasive tumours using pools of samples representing different stages (Thykjaer *et al*, 2001), or individual tumours (Dyrskjot *et al*, 2003; Sanchez-Carbayo *et al*, 2003a,2003b). The first attempts to apply DNA microarrays for prediction of recurrence (Dyrskjot *et al*, 2003) and survival (Dyrskjot *et al*, 2003; Sanchez-Carbayo *et al*, 2003b) have recently been reported. Many clinical questions remain to be evaluated by the application of DNA microarrays in classification, prediction of overall survival, recurrence, progression, or chemosensitivity to the current intravesical treatment in superficial disease or to the chemotherapy utilised in invasive and metastatic disease. It is expected that the more narrowed the clinical question to answer, the higher the number of well-annotated cases necessary for these analyses.

## FINAL COMMENTS AND REMARKS

Expression profiling using microarrays, as stated above, is not only changing both scientific and clinical paradigms, but is also advancing at a rapid pace. Technical advances and improvements are being made at each step of the microarray assembling line and analytical process. For example, the lower cost and wider variety of commercial arrays options, improvements in the speed and reliability, technologies of the spotting robots, improvements in probe labelling and hybridisation techniques, and changes in target design are just a few to mention. In a short period of time, DNA microarrays have moved from being a technology restricted to a few well-funded or technically sophisticated laboratories, to one that is widely used and will be incorporated into clinical laboratories. This trend will continue as the quality and ease of use of the technology increase and the costs decrease.

The studies summarised here and others indicate that expression profiling of a relatively small number of genes may provide a molecular means of identifying clinically important tumour subtypes and molecular targets not identified using standard methods. Moreover, these subtypes and targets may define subgroups of patients that will benefit from individualised treatment regimes. It is clear that the results of these studies will add to our understanding of the mechanisms of carcinogenesis and may also improve our ability to diagnose and treat bladder cancer. As interest in microarrays and their use in the study of cancer continue to increase, so does the likelihood that their use will have critical clinical applications.

**Figure 2:**
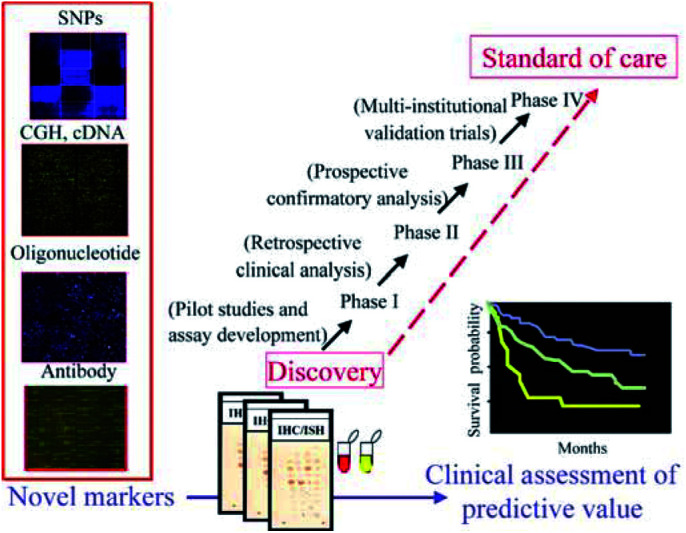
The use of high-throughput technologies provides novel targets that might be developed into biomarkers of diagnostic and prognostic utility. The process of development of a tumour marker requires several steps before acceptance into clinical routine.

Although the large amounts of gene expression data generated have already had a tremendous impact on bladder cancer research, major challenges remain. Microarray data can clearly improve tumour classification and provide empirical clinical correlations. However, the patterns and profiles observed may still be remarkably cryptic to answer specific clinical issues. Carefully controlled, large-scale expression profiling studies on large numbers of clinically well-annotated cases are needed before the final clinical utility of this technique can be accurately judged (Figure 2

).

Biologically straightforward results are emerging. Being able to link these data to oncogenic pathways, underlying mutational events, and genomic signals remains an important goal. This process must be reached in order to maximise insights into the underlying biology of cancer, which will ultimately prove to be of therapeutic relevance. Further approaches are required to combine data from tumour expression profiling with that obtained from model systems and genomic analyses, in order to link the complementary aspects of cancer biology.

Knowledge of the expression patterns of primary cancer cells, normal and malignant tissues is providing insights into the biology of bladder cancer. Global views of the malignant transcriptome and genome are proving to be valuable for understanding neoplastic transformation, cancer diagnosis, prediction of clinical outcome, and response to therapeutic intervention. Gene expression profiling could define, at the molecular level, the clinical and histopathological phenotypes of tumours. Moreover, the tumour subclasses are likely to reflect the basic differences in the cell biology of the tumours. Gene expression monitoring by DNA microarrays is becoming a novel tool for identifying new cancer classes (class discovery) or for assigning tumours to known classes (class prediction). Reports using different microarray platforms and analysing specific cancer subgroups are finding consensus on subclasses and signatures of the disease with predictive utility.

Overall, the use of microarray technologies for the study of bladder cancer remains a new research field. The results reported so far represent preliminary data that need to be contrasted by different groups using different series of patients. Creation of international tumour banks represents an option that might facilitate interactive research among different laboratories. Further efforts using *in vitro* and *in vivo* models are warranted to functionally characterise the pathways by which many of the targets are already identified to be involved in tumorigenesis or bladder cancer progression. The utility of the application of microarrays has not yet estimated many clinical issues. Identification of Ta-T1-IS subtypes within the superficial disease and patients more likely to develop positive lymph nodes or distant metastases are critical subclassification questions to be answered.

An area that will provide critical targets for clinical intervention is that of pharmacogenomics. Studies evaluating biological markers (at the DNA, RNA or protein level) to predict the drug efficacy or the relative risk of adverse effects in individual patients are still needed for many tumour types. In the near future, gene profiling will provide an effective means of predicting the response against specific therapeutic regimes based on the molecular signatures of the tumours associated with their chemosensitivity or resistance to anticancer drugs. Moreover, the discovery of molecular pathways altered in cancer progression, as well as the identification of molecule-susceptible targets, would lead to the development of novel alternative therapies. The combined information revealed by these studies allows also identification of new molecular determinants involved in the progression of the disease with clinical diagnostic or predictive utility. The classical tumour marker concept of an individual biological determinant will be substituted by the use of cluster of genes as predictive classifiers. These genetic signatures will allow a better chance of cure by opting for the most appropriate treatment, while maintaining the quality of life.
